# Advances in Porous Biomaterials for Dental and Orthopaedic Applications

**DOI:** 10.3390/ma3052947

**Published:** 2010-04-28

**Authors:** Meenakshi Mour, Debarun Das, Thomas Winkler, Elisa Hoenig, Gabriela Mielke, Michael M. Morlock, Arndt F. Schilling

**Affiliations:** 1 Department of Mechanical Engineering, National Institute of Technology, Durgapur 713209, India; E-Mails: meenakshi.mour@gmail.com (M.M.); debarun.das88@gmail.com (D.D.); 2 Biomechanics Section, Hamburg University of Technology, Hamburg D-21073, Germany; E-Mails: thomas.winkler@tuhh.de (T.W.); elisa.hoenig@tuhh.de (E.H.); gabriela.mielke@tuhh.de (G.M.); morlock@tuhh.de (M.M.M.)

**Keywords:** biomaterial, porosity, tissue engineering, bone, teeth

## Abstract

The connective hard tissues bone and teeth are highly porous on a micrometer scale, but show high values of compression strength at a relatively low weight. The fabrication of porous materials has been actively researched and different processes have been developed that vary in preparation complexity and also in the type of porous material that they produce. Methodologies are available for determination of pore properties. The purpose of the paper is to give an overview of these methods, the role of porosity in natural porous materials and the effect of pore properties on the living tissues. The minimum pore size required to allow the ingrowth of mineralized tissue seems to be in the order of 50 µm: larger pore sizes seem to improve speed and depth of penetration of mineralized tissues into the biomaterial, but on the other hand impair the mechanical properties. The optimal pore size is therefore dependent on the application and the used material.

## 1. Introduction: Bone and Teeth—Natural Porous Materials

### 1.1. Bone

The connective hard tissues bone and teeth appear to be made of solid materials at first glance; however they are highly porous on a micrometer scale. Trabecular bone has 50–90% porosity [1] and pore sizes in the order of 1 mm in diameter [2]. Even the solid structure of cortical bone has a series of voids, for example haversian canals, with a cross-sectional area of 2500–12,000 µm^2^ that results in 3–12% porosity [3]. These pores are filled with fluid and cells, making bone a viscoelastic material with remarkable regenerative capacity. This regenerative capacity is secured by four cell types that are present in the pores of bone tissue: osteoblasts, osteoclasts, osteocytes and bone lining cells. Vascular canals ramify within bone, providing its cells with metabolic support. In cancellous bone, the pores are also filled with red and yellow marrow. This bone marrow includes stem cells, which participate in the maintenance and organization of bone [4]. Despite the porous structure of connective hard tissues, they show high values of compression strength at a relatively low weight. Bone has a compression strength that is several times higher than that of concrete and due to its porous structure its density is only in the range of aluminium.

### 1.2. Teeth

Enamel, dentine, cementum, and pulp are the dental tissues. These four materials are joined at the cementum–enamel junction (CEJ) and dentine–enamel junctions (DEJ) [5]. The open porosity of the dental tissue varies between 1.11% and 3.08% of its volume [6]. While there are no cellular bodies present in enamel or dentin, the open pores in dentin are filled with Tomes’ fibers connecting the surface with odontoblasts lying within the teeth, suggesting an important function of the pores for tooth function. With a Mohs hardness of 5, enamel is harder than iron [7]. The fracture toughness of dentine is midway in the range observed for cortical bone and is at least one order of magnitude greater than the value for dentine-restorative materials.

### 1.3. Biomaterials

Unfortunately, caries, gnashing and osteoporosis lead to deterioration of bone and teeth, resulting in suffering of millions of patients, enormous costs for the societies and a great demand for orthopaedic and dental biomaterials. Implants have been extensively used in oral rehabilitation and orthopedics for replacements of lost or partially damaged skeletal tissues [4]. Ideal biomaterials for these applications need to simultaneously satisfy many requirements such as biocompatibility, strength, fatigue durability, non-toxicity, corrosion resistance, and sometimes aesthetics [8]. The wear characteristics and visco-elastic properties of implants are also important. In the case of bone, materials should preferably be both osteoinductive (capable of promoting the differentiation of progenitor cells down an osteoblastic lineage), osteoconductive (support bone growth and encourage the ingrowth of surrounding bone), and capable of osseointegration (integrate into surrounding bone) [9]. Nowadays, the strategy to design smart biomaterials lies in their capacity to instruct biological entities to entirely regenerate tissues; in other words, to create a synthetic twin tissue or organ that can function as its natural, original tissue. The Biomaterials field is shifting towards biologically “active systems” in order to improve their performance and to expand their use. Biomaterials as scaffold have been combined with autologous cells (*i.e.*, tissue engineering), to render tissue substitutes more ‘‘alive’’ and more reactive towards biological environment. More recently, there has been considerable interest in the development of ‘‘smart materials’’ that are able to instruct the behavior of adhered or encapsulated cells by releasing bioactive molecules into the local environment, or through extracellular protein/ peptide mimetics built into the delivery substrates [4]. A number of architectural characteristics including permeability, pore size and porosity play significant roles for this development.

### 1.4. Porosity

Porosity is defined as the percentage of void space in a solid and it is a morphological property independent of the material. Each porous material might have three types of pores: closed, through and blind pores [10]. The closed pores are not accessible to fluids. The blind pores terminate inside the material. The through pores are those that make possible the complete passageway of fluids. The open porosity includes only through and blind pores. Porosity that includes closed pores has a great impudence on mechanical properties of a material, open porosity has its direct impact in the possibility of penetration desired and undesired fluids, cells or bacteria. Porous metals with an interconnected pore structure are of particular interest for orthopaedic implant applications due to their potential ability to facilitate tissue ingrowth. Pores are necessary for tissue formation, because they allow migration and proliferation of cells, as well as vascularization. In addition, a porous surface improves mechanical interlocking between the implant biomaterial and the surrounding natural tissue, providing greater mechanical stability at this critical interface.

Approaches in scaffold design therefore often try to create hierarchical porous structures to attain desired mechanical function and mass transport (that is, permeability and diffusion) properties, and to produce these structures within arbitrary and complex three dimensional (3D) anatomical shapes. Mass-transport requirements for cell nutrition, porous channels for cell migration, and surface features for cell attachment necessitate a porous material structure.

## 2. Manufacturing Process for Porous Biomaterials

The fabrication of porous materials has been actively researched since 1943, when B. Sosnik attempted to introduce pores into aluminium by adding mercury to the melt [11]. In biomedical applications, the concept of using porous materials has been investigated much later: whereby the work of Weber and White from 1972 is one of the earlier to mention the use of porous metals for osseointegration [12]. Different processes vary in preparation complexity and also in the type of porous material that they produce. Thus, some processes such as casting or vapor deposition techniques tend to allow greater control over pore size, distribution and interconnectivity with open cell geometries. Interconnectivity of the pores is especially important for ingrowth of cells and their connection to the vascular system. Other processes involving decomposition of foaming agents in either molten or powder metal matrices give lower porosities and less predictable pore distribution and interconnectivity.

The most common techniques used to create porosity in a biomaterial are salt leaching, gas foaming, phase separation, freeze-drying and sintering depending on the material used to fabricate the scaffold [13]. Several techniques are nowadays available to create nanotopography on organic and inorganic surfaces, namely electron beam lithography, colloidal resists, self-assembling systems, casting, micro-contact printing, masters made by one of the above techniques and particle synthesis [14].

Depending on the designated application of porous materials, they can be grouped into three subgroups: porous coatings, physicochemically designed porous materials and rapidly prototyped porous materials designed with the aid of 3D-computer programs, as discussed in section 2.1, section 2.2 and section 2.3, respectively.

### 2.1. Porous coatings

Solvent casting, in combination with particle leaching, works only for thin membranes or 3D specimens with very thin wall sections: otherwise, it is not possible to remove the soluble particles from within the polymer matrix. The disadvantages of the this technology include the extensive use of highly toxic solvents, time required for solvent evaporation (days-to-weeks), the labor intensive fabrication process, the limitation to thin structures, residual particles in the polymer matrix, irregularly shaped pores, and insufficient interconnectivity [15].

Another commonly applied technique for the production of thin porous structures is plasma spraying. It can be used to create rough solid surface textures, porous surface coatings on solid cores and also fully porous structures. During plasma spraying, an electric arc is generated between two water-cooled electrodes in a gun. The arc heats the gas to extremely high temperatures (up to 20,000 °C), partially ionizing it and forming a plasma jet. The gases are accelerated by the tremendous expansion in volume and pass through the jet-shaped anode at a high speed. The powder for the coating is injected into the plasma gas stream, using a carrier gas where they are accelerated to a high speed, melted and impacted onto the substrate with high kinetic energy. Porous coatings with varying degrees of porosity can be created on the substrate by adjusting the spraying parameters. Plasma spraying is normally performed under vacuum where interactions between the plasma jet, powder, substrate and the surrounding atmosphere are reduced significantly [16].

Another variant of the process is reactive plasma spraying, where the starting powder materials are reacted with inert or reducing gaseous species and introduced into the plasma jet. Titanium plasma-sprayed coating is employed for fabricating porous-coated implants. Hahn and Palich used titanium hydride powders fed into plasma flame, whereby the decomposed titanium was deposited onto an appropriate substrate (titanium hydride starts to decompose at around 600 °C and reaches complete dissolution into titanium and hydrogen at 1000 °C). By choosing an appropriate gun-to-substrate distance, a thin coating (approximately 900 µm thick) with porosity that varied from zero at the substrate interface to about 50% at the coating surface was formed [17]. The formation of other metal surface coatings such as Co–Cr alloy, stainless steel or Ti–6Al–4V by this process is also possible. However, coatings prepared with this method result in irregular porosities and the pore interconnectivity is quite low compared to other techniques. Nevertheless, graded porous titanium coatings have been produced using plasma spraying and are characterized by a gradual change in porosity from the substrate-coating interface to the coating surface [18].

### 2.2. Physicochemically designed porous materials

Porous materials are classified as closed-cell and open-cell. In closed-cell foams each cell is completely enclosed by a membrane of the material, whereas in open-cell foams the individual cells are interconnected, allowing tissue to infiltrate the foam and anchor it into position. Closed-cell porous materials are usually the result of a random foaming process, in which the size, shape and location of pores within the matrix varies, depending on the parameters of the fabrication process [19].

#### 2.2.1. Closed-cell pores

There are two general routes to physicochemically generate closed porosity: melting and powder conditioning. In melting, self foaming structures are manufactured either by gas injection through the melt or by the addition of gas forming elements into the liquid material, usually metal. These methods have been used to fabricate Al, Zn and Mg foams; however, they are unsuitable for the manufacture of Ti foams, due to the high melting temperature and the associated reactivity of Ti with oxygen residues in the ovens. In powder metallurgy, the structures are obtained either by sintering hollow spheres or by melting of powder compacts that contain a gas evolving element such as TiH_2_ [20]. This approach has been known to yield a relatively homogenous structure and can be used in the manufacture of high melt metals and alloys. Fatigue strength can be improved by incorporating an adequate mixing strategy of the metal and foaming agent powders due to a resulting homogenous pore distribution. This helps minimizing stress concentrations within the structure and increasing fatigue life significantly [18]. The volume fraction of porosity is associated with the degree of particle interconnectivity and particle size. It can be controlled by process variables such as compacted powder density, sintering temperature and time, and alloying additions. The limitation of the powder sintering approach is that pore size and shape are dictated by the powder size and shape [21].

#### 2.2.2. Open-cell pores

The “Space Holder Method” is a fabrication process that can produce non-homogenous porous metal samples of greater porosity with an open cell structure. The process begins by mixing the metal powders with an appropriate space holder material and is followed by the compaction (e.g., uniaxial or isostatic) of the mix to form a green body. The resulting pellet is then subjected to a low-temperature heat treatment process that is designed to remove the space holder, which also leads to initial stage sintering of the metal particles that are in contact. The result is an initial neck formation. Continued sintering at higher temperatures further develops sinter neck growth. This leads to densification of the structure and associated improvement of structural integrity. This method provides a foamed structure with a close to homogenous pore structure and high levels of porosity (60–80%). By choosing the size, shape and quantity of the space holder used, the mechanical properties of the metal foam can be adjusted. Smaller sizes of the space holder particles can be obtained by sieving. A general difficulty of this method is the removal of large quantities of the space holder materials from the compacted mix [22].

“Replication” is an approach that is related to the above technique and uses a three-step procedure of pattern preparation, infiltration and pattern removal for the production of highly porous materials. Li *et al.* utilized this method to produce porous titanium and titanium alloy structures. Polyurethane foams were immersed in titanium slurry comprising Ti–6Al–4V powder (70 wt %), H_2_O (20 wt %) and ammonia solution. The ammonia solution was added to improve the rheological properties of the slurry. The sample was subsequently dried and the process was repeated until all the struts of the polyurethane foam were coated with Ti–6Al–4V powder. After thermal removal of the polyurethane scaffold and binder and subsequent sintering of the powders, a reticulated open-cell foam with hollow titanium struts remained. The rheological properties of the Ti slurry play an important role in the impregnation process, which is governed by the particle size and shape of the raw powder, the type and content of the binder, the solid/liquid ratio, pH, sedimentation behavior of the slurry and the amount of air bubbles in the mix. A rapid drying process is also important in maintaining a positive replica shape [23].

“Combustion synthesis” (CS) is a recently developed effective method for producing high purity porous alloys with non-homogenous pores, in particular nickel titanium alloys. In CS, particle fusion is obtained through an extremely rapid self-sustaining exothermic reaction driven by the large heat released in the synthesis. The reactants, in the form of fine powders, are usually dry-mixed and cold pressed. The exothermic reaction can then be instigated under two different regimes: (a) thermal explosion mode, in which the reactants are gradually heated until reactions take place simultaneously throughout the whole sample, and (b) self-propagating high thermal synthesis (SHS), which is characterized by the fact that once ignited, a strong exothermic reaction propagates as a combustion wave through the entire mixture, without requiring additional energy [24]. Various processing parameters such as the reactant particle size of powder, the use of a binder and the compaction pressure affect the final microstructure and porosity of the sample [25].

For the generation of large homogenous pores “Orderly oriented wire mesh” (OOWM) coatings for orthopaedic implants were created. Like the fiber mesh structures, this system makes use of small-diameter metal wires. However, rather than cutting these to short lengths for compaction, the continuous wire lengths are woven into a regular meshwork that is subsequently pressure sintered onto the solid substrate. The wire diameter, inter-wire spacing, and geometric distribution of the wires determine the dimensions of the interconnecting porosity [26].

“Vapor deposition” (VD) is a recently developed metallurgical technique attempted for creating metallic matrices of much greater porosity. Chemical vapor deposition (CVD) is the generic name for a group of processes that involve depositing a solid material on a substrate by activating the reactants in the gaseous phase where they react chemically [27]. Reactant gases, often diluted in a carrier gas, at room temperature enter the reaction chamber and the gas mixture is heated by radiation as it approaches the deposition surface or placed upon a heated substrate. Depending on the process and operating conditions, the reactant gases may undergo homogeneous chemical reactions in the vapor phase before striking the surface. Near the surface, thermal, momentum, and chemical concentration boundary layers form as the gas stream heats then slows down due to viscous drag, eventually changing the chemical composition. Heterogeneous reactions of the source gases or reactive intermediate species (formed from homogeneous pyrolysis) occur at the substrate surface forming the coating layer. Gaseous reaction by-products are transported out of the reaction chamber. This dictates the structural properties of the final product. The porosity (pore volume) of a typical trabecular metal component is approximately 75–85%, and is characterized by an average pore size (diameter) of between 550 µm [28].

Bone growth can be stimulated *in vivo* via a magneto-mechanical mechanism that involves the transmission of stresses and strains to growing bone via small local deflections within a porous ferromagnetic material, induced by the application of an external magnetic field. It has been determined that strain levels of at least about 1 millistrain (0.1%) are needed in order to stimulate bone growth. Utilizing this fact, Markaki *et al.* created implants with an outer layer of highly porous ferromagnetic fibers bonded together. The porous specimens were created by spraying a small quantity of fibers with a slow setting aerosol glue and then sprinkling some braze powder over them. The fibers, with braze particles adhering them, were packed into a long quartz tube and brazing was carried out at 1200 °C. Resulting specimens had porosity levels of about 75–90% and the fiber array had average pore sizes of 100–300 µm [29]. The primary requirement in the above technique is that the fiber material is ferromagnetic. It has been established that several such materials exhibit good corrosion resistance in biological fluids [30,31,32,33].

Apart from the above mentioned methods, there are also techniques that help produce functionally graded pore distribution. “Electrical field-assisted powder consolidation” known as field assisted consolidation technique (FAST) [34], spark plasma sintering (SPS) [35], plasma activated sintering (PAS) [36], and electrical discharge compaction (EDC) [37,38,39] combine electrical discharge with rapid heating and pressure application to achieve fast sintering of powders. Conventional sintering of Ti alloy powders requires maintaining a high sintering temperature (1200–1400 °C) in high vacuum (>10^-4^ Pa) for a long time (24–48 hours) [40]. This difficult sintering process limits the use of sintered Ti and its alloys. The aforementioned methods are useful in that they can easily sinter Ti and its alloy powders, because ionization in the plasma created by the high current discharge can melt the local oxide surface film on the particles, bringing the particles into contact with each other, and allowing junctions to be formed. Surface analysis performed by Lee *et al.* indicates that implants produced by this method allow rapid osseointegration [41]. Lifland *et al.* produced porous-surfaced Ti–6Al–4V implants using the same method. They found the compacts to have yield strengths ranging from 270 to 530 MPa and ultimate compressive strengths to range from 390 to 600 MPa [42]. Using SPS, Kon *et al.* produced porous Ti–6Al–4V with a porosity of 32% and compressive strength of 125 MPa [43].

Open-cell structures can also be fabricated by controlled sintering of powder performs [44] or solid-state foaming by superplastic expansion of argon-filled pores [45]. However, these manufacturing techniques have limitations concerning the control of the outer shape and the pore structure. Whang *et al.* developed a protocol for the fabrication of aliphatic polyester-based scaffolds by using the emulsion freeze-drying method. Scaffolds with porosity greater than 90%, median pore sizes ranging from 15 to 35 µm with larger pores greater than 200 µm were fabricated. The scaffold pore architecture was highly interconnected [46]. However, the emulsion freeze-drying method is user and technique sensitive. Nanotechnology led to promising novel approaches for manufacturing of porous scaffolds. One technique to obtain highly porous structures is electrospinning: Electrospun nanofibrous structures of poly (lactide-co-glycolide) had 92% porosity; the pore size distribution was broad (2–465 µm). In hydroxyapatite/chitosan-gelatin composites (with most pores between 300 and 500 µm) porosity can be increased by decreasing the chitosan-gelatin concentration and increasing the chitosan gelatin/hydroxyapatite ratio [47]. Recently an alternative method called equal channel angular pressing (ECAP) has been attempted to develop fine grain structure in grade 2 CP Ti. This process resulted in enhanced hardness, higher yield strength (increase by 140%) and higher fatigue strength (increase by 100%) compared to the coarse-grained materials [48]. L.Y. Yeo demonstrated the capability of the AC electrospray as a viable, safe and attractive alternative for micro/nano-encapsulation, bioscaffold production as well as polymeric nanoparticle fabrication over conventional fabrication techniques as well as DC electrospraying/ electrospinning [49]. The supercritical fluid-gassing process has been known for many years in the non-medical polymer industry [50] as well as in the pharmaceutical community [51]. This technology is used to produce foams and other highly porous products. The polymers which can be used for this technology have to have a high amorphous fraction. The supercritical fluid-gassing technology allows the incorporation of heat sensitive pharmaceuticals and biological agents. However, on average only 10-30% of the pores are interconnected [52]. Harris *et al.* combined this technology with particulate leaching to gain a highly interconnected void network. The researchers could control porosity and pore size by varying the particle/polymer ratio and particle size [53].

### 2.3. Rapidly prototyped porous biomaterials

For the design of complex scaffold architectures hierarchical image-based or CAD techniques are used, however, these designs cannot readily be built using the conventional techniques. Instead, scaffold architectures must be built using layer-by-layer for control over both global scaffold shape and 3D microarchitecture. These manufacturing processes are known collectively as solid free form fabrication (SFF). All SFF systems use a triangular facet surface representation of a structure, and build the 3D structure on a platform that moves to allow layering.

Most commercially available systems may be categorized into three major groups based on the way materials are deposited. The first group includes laser-based machines that either photopolymerize liquid monomer or sinter powdered materials. The second major group actually prints material, including printing a chemical binder onto powdered material or directly printing wax. The third major group is of nozzle-based systems, which process material either thermally or chemically as it passes through a nozzle. This class of systems include the Bioplotter, which is the only commercial machine developed to print biological cells as well as a range of biomaterials. Recently, Li *et al.* published a rapid prototyping technique to create porous Ti–6Al–4V implants with controlled size, pore shape and distribution using a three-dimensional (3D) printing of slurries. In this case the preparation of the slurry and the shrinkage of the porous structures after sintering seem to be critical steps [54,55].

Selective electron beam melting (SEBM) is a new additive manufacturing technique with high capability for the fabrication of porous metals with well-defined cellular structures. The basic principle of this technology is the generation of structures by the selective melting of discrete powder layers by an electron beam under vacuum. It was successfully used to fabricate novel cellular Ti–6Al–4V having diamond and hatched structures for orthopaedic applications [56]. Micro computer tomography (µCT) analysis demonstrated the capability to fabricate three-dimensional structures with an interconnected porosity and pore sizes suitable for tissue ingrowth and vascularization. Surface modifications were performed by a wet chemical treatment in HCl and NaOH. µCT measurements give a porosity of 80.5% and a mean pore size of 1.23 mm for the diamond structure, and 61.3% and 0.45 µm for the hatched structure, respectively [57]. Thus, the structures exhibit porosities comparable to that of trabecular bone.

Biomimetic porous scaffolds made of calcium phosphate mineral were designed with Computer Aided Design (CAD) software by Liulan *et al.* [58]. In order to fabricate the scaffolds, biomaterials, β-tricalcium phosphate (β-TCP), and polymeric blends were used on a selective laser sintering (SLS) system, a kind of rapid prototyping machine, to produce specimens. A versatile RP technique, selective laser sintering (SLS), offers good user control of scaffold microstructure by adjusting the process parameters. F.E. Wiria *et al.* used biocomposite material, consisting of poly-ε-caprolactone (PCL) and hydroxyapatite (HA), to fabricate tissue engineering scaffolds using SLS [59]. Hard tissue engineering scaffolds prepared by micro-extrusion freeforming using biphase calcium phosphate powders allows both the shape and a hierarchical pore structure within the shape to be designed on the computer and promptly downloaded onto a three-axis building platform [60]. Kalita *et al.* fabricated controlled porosity polymer-ceramic composite scaffolds, with 3D interconnectivity by high shear mixing of polypropylene (PP) polymer and tricalcium phosphate (TCP) ceramic by fused deposition process, one of the commercially available rapid prototyping (RP) techniques [61]. β-Tricalcium phosphate (β-TCP) scaffolds with designed, 3D geometry and mesoscale porosity have been fabricated by direct-write assembly (robocasting) techniques [62].

## 3. Analysis of Porosity in Biomaterials

To control the production processes of porous materials with different pore sizes, it is necessary to have methodology available for determination of pore properties. Different methods are used to measure porosity and pore size in scaffolds.

Total porosity (Π) is measured by gravimetry according to the equation [63,64]:

Π = 1 – ρ_scaffold_ / ρ_material_

Where, ρ_material_ is the density of the material of which the scaffold is fabricated and ρ_scaffold_ is the apparent density of the scaffold measured by dividing the weight by the volume of the scaffold.

Mercury intrusion porosimetry is a method used to measure both porosity and pore sizes [13]. The International Union of Pure and Applied Chemistry (IUPAC) has divided pores according to their diameter into three groups: micropores (D < 2 nm), mesopores (2 nm < D < 50 nm) and macropores (D > 50nm). The mercury porosimetry enables detection from macropores down to larger mesopores [13]. The scaffolds are placed in a penetrometer and infused with mercury under increasing pressure. As the pressure (P) increases, the radius of pores (r) that can be filled decreases according to the Washburn equation [64]:

P = 2σ cos Ө/r

Where, σ is the surface tension of mercury and Ө is the contact angle. The open porosity (π) (porosity accessible to mercury intrusion) is given as [64]:

**π** = **V**_intrusion /_**V**_scaffold_

Where, V_intrusion_ is the total intrusion volume of mercury and V_scaffold_ is the volume of the scaffold. The open porosity can be calculated by the liquid displacement method as well. The scaffold is submerged in a known volume V_1_ of liquid that is not a solvent for the scaffold and a series of brief evacuation–repressurization cycles is conducted to force the liquid into the pores of the scaffold. After these cycles, the volume of the liquid and liquid-impregnated scaffold is V_2_.

When the liquid-impregnated scaffold is removed, the remaining liquid volume is V_3_ and open porosity is given as [65,66]:

π = (V_1_­ V_3_) / (V_2_ – V_3_)

Images of scanning electron microscopy (SEM), transmission electron microscopy (TEM), accompanied with energy and wave dispersive X-ray microanalysis are analyzed with various computer software to measure porosity and, particularly, pore sizes [13]. For statistical analysis, different sample sizes are used, for example, ranging from 10 to 40 pores [65,67] to a maximum of 100 [68]. Finally, micro computed tomography (µCT) imaging and analysis have been used to determine porosity and pore sizes in 3D biomaterial scaffolds used in bone tissue engineering [69,70]. Briefly, isotropic slice data are obtained and reconstructed into 2D images, which are compiled and analyzed to generate 3D images to obtain quantitative morphological detail [70]. This technique is particularly appealing, since it is non-invasive and can be used to image and quantify bone repair. Synchrotron radiation-based 3D-microtomography (SRμ-CT) is a comparatively new method that permits the reconstruction of internal structures of millimeter-sized objects within micrometer resolution [71]. This family of methods also includes scanning tunneling microscopy, (STM) (tunneling current contrast) and atomic force microscopy (AFM) (interatomic force contrast) [72]. In practice, AFM has the advantage over more traditional methods such as in that surfaces can be measured in the presence of hydration or even liquid water *in vivo*, and there is no need for staining as in the optical microscopies, or even worse, the high vacuum and conductive films needed for SEM studies [73].

The elastic modulus, E, of the porous composite is given by [74]:

E = E_o_ [1 – (P + V_GP_)] ^n^

Where, (E_o_) is the elastic modulus of the solid composite, (P) the pore fraction, (V_GP_) the glass fraction incorporated in the pore space and (n) a constant that depends on the microstructure.

The rapidly increasing use of ultrasound to detect and monitor bone pathology, in particular osteoporosis, has led to the development of several commercially available devices. Such devices typically measure the rate of change of attenuation of ultrasound with frequency (broadband ultrasound attenuation or SUA) between 0.2 and 0.6 MHz, and also the velocity of sound to compute the porosity [75].

## 4. Porous Biomaterials in Orthopaedic and Dental Applications

### 4.1. Cell reaction to porous structures

It has been demonstrated that cells can react *in vitro* to objects as small as 5 nm, which are 1000–5000 fold smaller in size than the cell itself [76]. Microporosity results in larger surface area that is believed to contribute to higher bone-inducing protein adsorption as well as to ion exchange and bone-like apatite formation by dissolution and re-precipitation. Surface roughness enhances attachment, proliferation and differentiation of anchorage-dependent bone forming cells [77]. Rough apatitic surfaces appear to enhance osteoclastic attachment compared with smooth ones [78]. Grooved surfaces influence osteoblast guidance, as does the groove profile and topography, independent of the chemical nature of the substrate [79]. On the other hand, on micro and macroporous calcium phosphate ceramics, osteoblasts sense the surface micro-porosity and can bridge even large pores many times larger than fully spread osteoblasts [80]. *In vitro* microfeatures with specific shapes can influence the cellular activities, including osteogenic differentiation [81]. *In vivo*, the surface micro topography can significantly affect tissue neo-formation. For example, the initial surface roughness of the titanium prostheses greatly influences early bone formation and contact with the implant [82,83]. D’Lima *et al.* showed that surface roughness was more important for osseointegration of titanium implants in rabbit femors, since an acid-etched coating (highest surface roughness) showed a higher overall osseointegration when compared with grit-blasted and fiber mesh (average pore size 400 µm) coatings [84]. The presence of a thicker (600–1000 nm) porous (13–24% porosity, pores less than 8 µm) oxide film on the surface of titanium screws resulted in more bone formation when implanted in tibia defects in rabbits compared to controls with a nonporous oxide layer of 17–200 nm in thickness [85,86]. Lower porosity of the oxide layer (19% *versus* 24%) resulted in decreased surface roughness (0.97 *versus* 1.02 µm) as measured by confocal laser scanning profilometry [87]. Other reports on the cellular interactions with specific nano-patterned substrates of various compositions have shown that nano-shaped holes can also (i) control cell life and death [88] and (ii) orient cell commitment towards osteogenic lineage [89,90]. Park *et al.* found that on a TiO_2_ nano-tube surface, a lateral spacing geometry with openings of 15–50 nm enhances the cell adhesion and spreading [91]. Arnold *et al.* proposed that the separation of 58–73 nm between the gold nano-dots is a universal length scale for integrin clustering and activation, leading to a better cell attachment and spreading [92]. Demirel *et al.* studied the fibroblast cell attachment and growth on nano-engineered sculptured thin films. They found that nanoscale topography, especially when compared with flat surface, enhances the cell adhesion of fibroblast cells [93]. The nano/submicron-scale TiO_2_ network layer (lateral pore size: 20–160 nm) significantly enhanced the whole blood coagulation and human bone marrow stem cells adhesion on the anodized Ti surface for dental implant application [94]. The influence of surface nano-topography on cell behavior is mediated via changes in the orientation and conformation of proteins that interact with the nanotextured substrate. Porous solid structures allow a strong adhesion of cells onto the surfaces. The asymmetry and the presence of concavities may increase the wettability of the substrate, and therefore enhance cell adhesion and survival.

### 4.2. Bone healing, ectopic bone formation and bone cell support by porous biomaterials

A variety of animal studies using different pore sizes and distributions showed healing of bone defects with the help of porous materials [17,95,96,97,98,99,100,101,102,103,104]. Cylindrical synthetic porous hydroxyapatite implants with pore sizes of 400–600 µm and 80% porosity healed femoral defects in rats [105]. Porous particles of hydroxyapatite (average pore size 150 µm, porosity 70%) and porous coral-replicated hydroxyapatite (exo-skeletal microstructures of calcium carbonate of corals converted into hydroxyapatite by hydrothermal chemical exchange) blocks (average pore size 230 µm, porosity 66%) were used for delivery of BMP-2 in a rat ectopic model and induced direct osteogenesis (without preceding cartilage formation) [106]. Other types of ceramics used in bone repair include porous calcium metaphosphate ([Ca (PO_3_)_2_]_n_) blocks (pore size 200 µm) that were used for culturing rat marrow stromal cells ex vivo and for ectopic bone formation in athymic mice [107] and natural coral scaffolds moulded into the shape of a human mandibular condyle with pore sizes 150–220 µm and 36% porosity that were seeded with rabbit marrow mesenchymal cells and induced ectopic bone formation in nude mice [108]. Combinations of ceramics also have been explored: porous biphasic ceramic (hydroxyapatite—tricalcium phosphate) with 50% porosity and 100–150 µm pore sizes have been shown to heal femoral defects in dogs [109]. Brittleness and slow degradation rates are disadvantages associated with their use. Gong *et al.* fabricated glass implants with 5% porosity and pores that ranged from 100–200 µm to the <10 µm range, and also glass-ceramic implants with macropores (100–200 µm) and micropores (<5 µm) [110]. Glassy carbon pellets with 40% porosity induced bone in-growth in tibia defects in rabbits [111]. Bioglass (materials with different compositions of SiO_2_, CaO, Na_2_O, andP_2_O_5_ [106,107] ) scaffolds have been shown to support culture of human primary osteoblasts [112] and have an interconnected network, 10–500 µm, and framework (2–50 nm) [107]. In other studies, Bioglass implants with pores ranging from 100 to 600 µm induced ectopic bone formation in dogs [114]. Silica/calcium phosphate scaffolds with different porosities (51%, 47% and 43% generated by decreasing the silica content) and a broad distribution of pore sizes (10–300 µm) helped to regenerate bone in femoral defects in rabbits. Upon retrieval, the silica-rich scaffolds were almost filled with new bone and showed higher restorability than scaffolds with lower silica content [115].

### 4.3. Biological effects of higher and lower porosity

Higher porosity should result in increased cell proliferation, since pore space increases with porosity and facilitates transport of oxygen and nutrients. To test this hypothesis, using a solid free form fabrication technique a porosity gradient from 80% to 88% was created in scaffolds of poly(l-lactide-co-d,l-lactide) containing 20 wt % b-tri-calcium phosphate (pore size 125–150 µm). Indeed, more tissue ingrowth and new bone formation occurred in areas with higher porosity after implantation in rabbit craniums. Scaffolds formed with four axial and four radial macroscopic channels also enhance osteogenesis [116]. Dental implants were coated with cancellous structured titanium with 44% and 48% porosity and implanted in canine mandibles and femorals; there was more bone ingrowth for the higher porosity coatings at all time points (14 weeks) in the femorals and at the initial time points (two and four weeks) in the mandibles [117]. While comparing hydroxyapatite scaffolds with different porosities [70% porosity and 800 µm average pore size (70/800) *versus* 60% porosity and 400 µm average pore size (60/400)] Kruyt *et al.* found that when scaffolds seeded with goat bone marrow stromal cells (gMSC) were implanted in bilateral paraspinal muscles in goats, more bone formed in the 70/800 scaffolds [118]. This result was likely due to the larger surface area that resulted in higher ion exchange and bone-inducing factor adsorption. The necessity for porosity in bone regeneration has been shown by Kuboki *et al.* using a rat ectopic model and solid and porous particles of hydroxyapatite for BMP-2 delivery: no new bone formed on the solid particles, while in the porous scaffolds direct osteogenesis occurred [106]. Titanium fiber-metal porous coatings (45% porosity and 350 µm average pore size) maximized bone ingrowth and increased the potential for stress-related bone resorption of femoral stems in a canine total hip arthroplasty model [119]. A similar result was observed for plasma spray-coated titanium implants with 56–60% porosity, although bone ingrowth was maximized for an open-pore titanium fiber mesh (60% porosity and 170 µm average pore size) coated with polyvinyl alcohol hydrogel [120]. Thus, due to the absence of any substantial report on the beneficial effects of lower porosity scaffolds *in vivo*, we can safely conclude that highly porous implants facilitate tissue integration.

### 4.4. Mechanical properties of porous biomaterials

However, the trade-off of better biological properties due to higher porosity is diminished mechanical strength, which defines a practical upper limit for pore size and porosity. Initial stress concentrations at pores decrease ﬂexural strength, lower resistance to fatigue, and increase wear [121,122]. Studies have shown that both Co–Cr alloys and Ti–6Al–4V alloys experience drastic reductions in fatigue strengths when fabricated as porous coatings on solid core structures [123,124,125,126]. It has been shown that the high cycle fatigue strength of porous coated Ti–6A1–4V alloy is approximately one-third that of the solid alloy equivalent shape, probably even less in fully porous matrices [127]. The bond sites between the coatings and implant have irregular geometries that can act as stress concentrations. This is sometimes referred to as the notch effect. This notch effect is a localised condition that affects implant strength in the region of the porous coating [123]. Cook *et al.* showed that an approximately 15% improvement in fatigue properties of porous Ti–6Al–4V could be achieved through post-sintering heat treatments that produce microstructures that are more resistant to crack initiation and propagation [128]. Ishikawa and Asaoka concluded that pressurized curing increases mechanical strength of calcium phosphate cements by decreasing porosity [129]. James *et al.* reported that all fatigue cracks in PMMA initiated at internal pores and that porosity, pore size, and pore size distribution affected the crack initiation and fatigue behavior [130]. Interfacial integrity between particles and matrix is the key for good mechanical properties. Sunnegardh *et al.* observed a similar problem for calcium aluminate cement [131]. Its heterogeneous microstructure and surface porosity limited its polishability, compared to resin composite and polyacid-modiﬁed resin composite [132]. Higher porosity (80% as opposed to 58%) decreased mechanical properties of porous poly(l-lactide-co-d,l-lactide) scaffolds: compressive strength decreased from 11.0 to 2.7 MPa and modulus from 168.3 to 43.5 MPa [133]. Increasing the pore size from 45–150 to 300–600 µm increased the elastic modulus (3.1–7.8 MPa) but did not affect yield strength in scaffolds produced by photopolymerization of a multifunctional lactic acid-based oligomer created by grafting 10 lactic acid units on each side of a di(ethylene glycol) core [134]. The porosity of these scaffolds was 80%, since lower porosity resulted in less interconnected pores [134] and higher porosity to scaffolds with low mechanical properties [133]. Eighty percent porosity was the critical point between inter-connectivity and mechanical properties of scaffolds made by photo-crosslinking of poly(propylene fumarate) as well; the toughest scaffolds with fully inter- connected pores fabricated by this technique had an elastic modulus of 2.3 MPa and a compressive strength of 0.11 MPa [135]. Although higher molecular weight (1.45 kDa) poly(propylene fumarate) increased the fracture toughness (0.376 MPam1/2 as opposed to 0.134 MPam1/2 for the 0.86 kDa) of scaffolds coated with b-tricalcium phosphate with pore sizes 150–300 µm, it reduced the porosity (69% compared to 74%) [130]. Hydroxyapatite powder has been sintered to generate blocks with fully interconnected pores (500 µm), 77% porosity, compressive and three-point bending strength of 17.4 MPa and 7.2 MPa, respectively, and elastic modulus of 0.12 GPa. These scaffolds induced ectopic bone formation when implanted subcutaneously in mice. Zhang *et al.* discussed a computational model to predict the effect of porosity on the mechanical proper- ties of poly(L-lactide)/bioactive glass composites with pores between 50 and 200 µm present in a network of smaller interconnected pores (<10 µm) [74].

Implant stability is not only a function of strength, but also depends on the fixation established with surrounding tissues. A major problem concerning metallic implants in orthopaedic surgery is the mismatch of Young’s modulus between bone (10–30 GPa) and bulk metallic materials (between 110 GPa for Ti and 230 GPa for Co–Cr alloys). Due to this mechanical mismatch, bone is insufficiently loaded and becomes stress shielded, which eventually leads to bone resorption [137,138,139]. Porous metals represent a promising means of reducing stiffness mismatch and avoiding stress shielding effects. To overcome the mechanical limitations of porous materials, novel composite materials have been investigated. Chitosan sponges with 100 µm pores were formed inside hydroxyapatite/b-tricalcium phosphate scaffolds with macropores (300–600 µm) and both compressive modulus and yield stress increased about four times [140]. Coating hydroxyapatite scaffolds (87% porosity and 150–200 µm pore size) with a hydroxyapatite/ poly (e-caprolactone) composite improved the mechanical properties: higher amounts of the composite coating (more polymer) increased compressive strength (maximum 0.45 *versus* 0.16 MPa for no coating) and elastic modulus (maximum 1.43 *versus* 0.79 for no coating) [141]. Collagen scaffolds have been coated with hydroxyapatite (pores 30–100 µm, porosity 85%), since osseointegration is enhanced by the surface formation of a bioactive apatite layer and this layer supported attachment and proliferation of rabbit periosteal cells [136]. Coating porous-surfaced titanium implants (35% porosity and 50–200 µm pore size) with calcium phosphate resulted in earlier and greater bone ingrowth and enhanced mechanical properties for implants retrieved from rabbit femorals [143]. Studies made on the correlation between the superelasticity behavior, the different pore size and various heat treatment conditions of NiTi produced by gas expansion method revealed that the NiTi with 16% porosity exhibited excellent combination of mechanical properties such as high strength (1000 MPa), low young modulus (15 GPa), large compressive ductility (>7%), large recoverable strains (>6%) and high energy absorption (>30 MJ/m^3^) [144].

### 4.5. Minimal necessary pore dimensions

Studies trying to determine the minimal necessary pore diameter for bone ingrowth found no significant difference in bone growth for 500 μm and 1,600 μm pores for PLGA scaffolds made by a 3D printing technique [116,145]. HA scaffolds with pore diameters ranging between 400 μm and 1,200 μm in a minipig mandibular defect model and HA scaffolds with 300 μm and 800 μm to deliver human gingival fibroblasts was transduced with BMP-7 in a mouse model. They found significant bone growth on designed scaffolds for all pores, with no statistical difference between pore sizes. This contrasts results using non-SFF scaffolds, where optimal pore diameters ranging from 200 μm to 600 μm have been suggested. However, unlike the single pore diameter in the designed scaffolds, non-designed scaffolds have a range of pore sizes, which may explain the different results [145]. A recent review established that the minimum requirement for pore size is considered to be approximately 100 µm. The minimum pore size required to regenerate mineralized bone is generally considered to be 100 µm due to cell size, migration requirements and nutrient transport [13]. In a study by Hulbert *et al.* calcium aluminate cylindrical pellets with 46% porosity with 100 µm were implanted in dog femorals. Large pores (100–150 and 150–200 µm) showed substantial bone ingrowth. Smaller pores (75–100 µm) resulted in ingrowth of unmineralized osteoid tissue. Smaller pores (10–44 and 44–75 µm) were penetrated only by fibrous tissue [146]. These results were correlated with normal haversian systems that reach an approximate diameter of 100–200 µm. However, using laser perforation techniques and titanium plates, four different pore sizes (50, 75, 100 and 125 µm) were tested in rabbit femoral defects under non-load-bearing conditions [147]. Bone ingrowth was similar in all the pore sizes. When primary rat osteoblasts were seeded into scaffolds with different pore sizes, more cells were found in the small pore (40 µm) scaffolds [146]. Similarly, smaller pores (0.4 and 13 µm) in TiO_2_ films on titanium surfaces enhanced the proliferation of human cells trypsinized from bone in contrast to larger pores (49 µm) [148]. Increasing pore size from 35.4 to 45.7 µm by decreasing the initial percentage of hyaluronate polymer (10% *versus* 66%) in collagen/hyaluronate scaffolds resulted in more new bone formation in rat calvarial defects [149]. These studies suggest that at least under not load-bearing conditions the minimum pore size permissive for bone ingrowth might be close to 50 µm.

### 4.6. Optimal pore sizes for bone

Apart from the work investigating the minimum requirement of pore size, many researchers have explored pore sizes above 100 µm in order to define optima for bone-related outcomes. Porous blocks of hydroxyapatite with different pore sizes (106-212, 212–300, 300–400, 400–500 and 500–600 µm) were compared when implanted subcutaneously in rats [150,151]. Alkaline phosphatase activity, osteocalcin content and new bone formation were higher for the 300–400 µm pore size. Onset of bone remodeling was delayed in surface laser-textured titanium alloy (Ti6Al4V) with 100 µm pores *versus* implants with 200 and 300 µm pores that were implanted in distal femoral cortex of rabbits [150]. Although the 300 µm pore implants had the highest percentage of lamellar bone, their osseointegration was slower than the 200 µm pore size implants based on the lower percentages of total (within-pore and surface bone-implant) contact. Hydroxyapatite scaffolds with small (90–120 µm) and large tunnel (350 µm) diameters were used for BMP-2 delivery and were implanted subcutaneously in rats [150,153,154]. In small diameter tunnels chondrogenesis occurred before osteogenesis; in contrast, in tunnels with large diameter bone was formed directly. The enhanced vascularization that was observed in tunnels with the larger diameters resulted in higher oxygen tension and supply of nutrients, conditions that favored direct osteogenesis.

### 4.7. Porous materials in dental applications

While it has been demonstrated that porous materials can be used to establish an effective means of implant stabilization by tissue ingrowth, it has been shown that extreme caution should be exercised in applying this concept to dental implants placed perimucosally. The surface microporosity that is adjacent to the gingival cuff results in an inflammatory reaction that prevents formation of an effective biological seal. Clinical failure soon follows. Observations show that an effective biological seal cannot be established with materials possessing crown and cervical surface microporosity [155].

Another problem in dental application of porous biomaterials is the aggressive chemical environment in the mouth, due to the greater availability of oxygen and acidic food stuffs, favoring corrosion. Increased surface areas, such as in porous implants, have shown higher corrosion rates when tested *in vitro* compared to conventional nonporous-coated implants [156,157]. Corrosion can severely limit the fatigue life and ultimate strength of the material, leading to the mechanical failure of the implant. There is a low but definite prevalence of corrosion-related fracture of implants [158]. Pitting corrosion of cobalt based alloys leads to the release of carcinogens into the body [159,160,161]. Enhanced metal ion release could increase the probability of metal sensitisation and associated allergic responses in individuals could increase the susceptibility to tumour formation. Pitting corrosion is a common problem with 304 SS implants. Introduction of ultra-high clean grades such as 316LVM and nitrogen additions have reduced the risk of pitting corrosion. Though titanium and its alloys are highly resistant to pitting corrosion in different *in vivo* conditions encountered, they undergo corrosion in high fluoride solutions in dental cleaning procedures.

## 5. Future Prospective and Outlook

### 5.1. Dental materials

Investigation into the effect of filler on dental material properties would be beneficial in the development of restorative dental material [162]. Further work is required to devise a method of reducing flaws and initiation of micro-cracks, and means of creating stronger interfacial bonding [163]. A topic in the research field of joining that needs to be addressed is the cementation of dental restorations on natural teeth or implants. This part of the restorative procedure is vital to the successful application of biomaterials in dentistry and the development of improved adhesive systems or the utilization of biological mechanisms. Furthermore, suitable materials must be developed for fabricating abutments similar to those used in conventional metal systems [164].

### 5.2. Bone tissue engineering

The challenge in tissue engineering bone and cartilage is not only to design, but also to fabricate reproducible bioresorbable 3D scaffolds, which function for a certain period of time under load-bearing conditions [165]. Controlling the degradation kinetics of biomaterials to match tissue growth, to create space for the new tissues to grow until full regeneration is reached remains a challenge in biomaterial design. One of the basic problems from a scaffold design point of view is that to achieve significant strength the scaffold material must have sufficiently high inter-atomic and inter-molecular bonding, but must have at the same time a physical and chemical structure which allows for hydrolytic attack and breakdown [165]. A number of textile technologies have the potential to design and fabricate highly porous scaffolds. Yet, only so-called non-woven mesh-like designs have been used to tissue engineer bone and cartilage. Excellent results in tissue engineering cartilage have been achieved by using non-woven meshes composed of polymer fibers of PGA, PGA/PDLA, and PGA/PLLA [166]. The potential to combine 3D printing of scaffolds with 3D printing of cells and biologics, while currently challenging, may enable the development of new designer materials/biofactors hybrids. Soft material routes like sol-gel processing might also be a strategy to incorporate biomolecules during scaffold fabrication, although this is still under development [9]. In the body, bone often has a structurally important interface with other tissues such as cartilage and ligament/tendon, for which designed scaffolds can be used to create tissue interfaces.

### 5.3. Interfaces

Surfaces are the primary place of contact between a biomaterial and its host organism. Typically, prostheses have to fulfill demanding structural and mechanical requirements, yet the material best for those functions may be bio-incompatible. Surface treatment or coating provides a means to overcome that problem, which means both integration within the host physiology and stabilization with respect to corrosion and wear. The adsorption of bio-macromolecules is pivotal for biocompatibility. The impossibility of keeping proteins away from most implants means that very careful consideration has to be given to this aspect, and both prevention (for bloodstream implants) and promotion (for bone replacement and repair) occur with equal importance [167]. Further elucidation of the communication between cells and of the complex interplay between cells and their matrix will help focus strategies to enable the presentation of biofactors in the correct context both chemically, temporally, and in terms of their distribution. Similarly, the clinical application of surface structuring approaches will require further understanding of the interactions occurring at the cell surface/substrate interface [9].

### 5.4. Chemical/materials systems of porous biomaterials

Surface reactivity is one of the common characteristics of bone bioactive materials and especially important in porous biomaterials because of the much bigger surface area of porous structures. During implantation, reactions occur at the material–tissue interface. That leads to time-dependent changes in the surface characteristics of the implant material and the tissues. An ion-exchange reaction between the bioactive implant and surrounding body fluids can results in the formation of a biologically active carbonate apatite (CHAp) layer on the implant that is chemically and crystallographically equivalent to the mineral phase in bone. This can contribute to the bone bonding ability of the material and have an enhancing effect on bone tissue formation [168]. BS ISO 23317 is the international standard that describes the method for detecting apatite formed on a surface of a material in simulated body fluid (SBF) with ion concentrations nearly equal to those of human blood plasma. The formation of apatite layers can be detected by thin film X-ray diffraction spectrometry and/or scanning electron microscopy. The apatite formed has a similar composition and structure to bone mineral. The evaluation of apatite-forming ability on implant material in SBF is useful for evaluating its ‘*in vivo*’ bone-bonding ability preliminary to animal experiments. ‘*In vitro*’ calcification could be shown on surfaces of Bioglass®, CaO-SiO_2_ glasses, Na_2_O-CaO-SiO_2_ glasses, Cerabone® A-W, Ceravital®-type glass-ceramic, sintered hydroxyapatite and alkali-heat-treated titanium metal, and is correlated with ‘*in vivo*’ calcification. However, this does not exclude the possibility that materials, which do not form apatite on their surfaces ‘*in vivo*’, bond to living bone. For example, it has been reported that resorbable materials as beta-tricalcium phosphate (Ca_3_(PO_4_)_2_ ) and calcium carbonate bond to living bone without forming an apatite layer on their surfaces.

### 5.5. Pore architecture

Engineering pore distributions to match the mechanical properties of bone is commonly accepted to be the next major improvement in the design of open-cell porous materials. However, the question remains as to how to identify the optimal position, shape, density and size of pores. The answer to this might lie in simply reconstructing the structure of trabecular bone from the anatomical site into which the porous material is to be implanted, based on the use of µCT images. An alternative method of obtaining information that could be used in engineering the parameters of porous materials might be the precise mapping of loads that occur in bone and teeth during various activities of daily living and to use that information as a design input information.

### 5.6. Nanotechnology

Most of the current design/fabrication occurs at scales above 100 µm, future work surely will strive to incorporate micrometer and nanoscale features. Currently, integration of micrometer or tens of micrometer feature sizes occurs during post-processing steps [165,169,170]. Integration of micrometer and nanoscale features into designed scaffolds might improve both mechanical properties through toughening mechanisms and tissue regeneration through improved control of cell adhesion. However, near-term advances in this area will probably occur through post-processing or a combination of nanofabrication techniques with indirect SFF.

Advances in materials processing are having a positive impact on the field of biomaterials today. It will be exiting to follow, how actual technologies and novel ideas in this field will further improve our possibilities of designing implants for people with diseases of the hard connective tissues.
